# Acid-sensing ion channel 3 mediates peripheral anti-hyperalgesia effects of acupuncture in mice inflammatory pain

**DOI:** 10.1186/1423-0127-18-82

**Published:** 2011-11-09

**Authors:** Wei-Hsin Chen, Ching-Liang Hsieh, Chun-Ping Huang, Tzu-Jou Lin, Jason TC Tzen, Tin-Yun Ho, Yi-Wen Lin

**Affiliations:** 1 College of Agriculture and Natural Resources, Graduate Institute of Biotechnology, National Chung Hsing University, Taichung, Taiwan; 2 College of Chinese Medicine, Graduate Institute of Acupuncture Science and Acupuncture Research Center, China Medical University, Taichung, Taiwan; 3 College of Life Sciences, Department of Life Sciences, National Chung Hsing University, Taichung, Taiwan; 4 College of Public Health, Department of Occupational Safety and Health, China Medical University, Taichung, Taiwan; 5 College of Chinese Medicine, School of Chinese Medicine, China Medical University, Taichung, Taiwan

## Abstract

**Background:**

Peripheral tissue inflammation initiates hyperalgesia accompanied by tissue acidosis, nociceptor activation, and inflammation mediators. Recent studies have suggested a significantly increased expression of acid-sensing ion channel 3 (ASIC3) in both carrageenan- and complete Freund's adjuvant (CFA)-induced inflammation. This study tested the hypothesis that acupuncture is curative for mechanical hyperalgesia induced by peripheral inflammation.

**Methods:**

Here we used mechanical stimuli to assess behavioral responses in paw and muscle inflammation induced by carrageenan or CFA. We also used immunohistochemistry staining and western blot methodology to evaluate the expression of ASIC3 in dorsal root ganglion (DRG) neurons.

**Results:**

In comparison with the control, the inflammation group showed significant mechanical hyperalgesia with both intraplantar carrageenan and CFA-induced inflammation. Interestingly, both carrageenan- and CFA-induced hyperalgesia were accompanied by ASIC3 up-regulation in DRG neurons. Furthermore, electroacupuncture (EA) at the ST36 rescued mechanical hyperalgesia through down-regulation of ASIC3 overexpression in both carrageenan- and CFA-induced inflammation.

**Conclusions:**

In addition, electrical stimulation at the ST36 acupoint can relieve mechanical hyperalgesia by attenuating ASIC3 overexpression.

## 1. Background

Inflammatory pain is crucial, disabling, and difficult to treat clinically. It includes both cell and non-cell immune inflammatory components. This type of pain is usually associated with peripheral tissue damage, ischemia, hypoxia, acidosis, and inflammation [1,2]. Tissue injury always results in inflammation that is often accompanied by sensitization of nerve terminal nociceptors innervating into the injury site. It is often initiated by a complex signaling to activate a complex response which alters the excitability of sensory neurons [3,4]. Chronic inflammation of injured regions usually increases the expression of inflammatory mediators such as cytokine, interleukin, proton, histamine, bradykinin, prostaglandin, and mast cells to activate immune cells and nerve terminals [5]. These mediators can bind to their specific receptors and further activate nociceptors to enhance the neuronal transduction to deliver pain signals.

Inflammation pain can be separated into primary hyperalgesia and secondary hyperalgesia. Recently, an animal model of inflammation pain was induced by injection of carrageenan or CFA into the peripheral tissue to induce both primary and secondary hyperalgesia [6,7]. By definition, an enhanced nociceptive response to noxious stimuli in an injury site is defined as primary hyperalgesia. For instance, mechanical hyperalgesia of the knee was initiated with carrageenan injected into the knee joint [8]. In contrast, an increased nociceptive response to noxious stimuli outside of the injured area is always considered as secondary hyperalgesia. For example, mechanical and heat hyperalgesia of the paw was observed in mice after carrageenan inflammation in the muscle [9]. Inflammation can sensitize nerve nociceptors both in peripheral and central nerve systems. This can enhance neuronal excitability and increase responses to mechanical stimuli following inflammation [10,11]. Dorsal root ganglion (DRG), the spinal cord dorsal horn (SCDH), and brain neurons show increased nociceptive receptors and a decreased threshold to noxious stimulation during inflammation [12].

Many ion channels and receptors participate in inflammation pain, including acid sensation ion channels 3 (ASIC3), transient receptor potential vanilloid 1 (TRPV1), voltage dependent sodium channel (Nav), and calcium channels [13]. Several inflammatory reagents such as carrageenan, kaolin, and Complete Freund's adjuvant (CFA) have been widely used in pain investigation. Carrageenan is often used to produce non-immune-mediated inflammation [14]. Injection of carrageenan into the paw or gastrocnemius muscle (GM) can induce inflammatory responses with an increase of mast cells, neutrophils, and macrophages. CFA is constituted of an antigen solution with heat inactivated *Mycobacterium tuberculosis *to potentiate the cell-mediated immune response and inflammation [15]. Microinjection of CFA into the plantar paw or GM can evoke persistent inflammatory hyperalgesia [7,16].

ASIC3 is mainly expressed in peripheral sensory neurons and is the most sensitive channel for acid detection [17]. ASIC3 can be activated within a narrow range of acidic pH (7.2-6.9) and enhanced by arachidonic acid and lactate [18,19]. ASIC3 participated in mechanical but not heat hyperalgesia after carrageenan-induced inflammation of the paw and repeated acid injection induced chronic pain [7,20]. Four genes encode 7 subtypes of receptors: ASIC1a, ASIC1b, ASIC2a, ASIC2b, ASIC3, ASIC4, and ASIC5. All of the ASIC superfamily are expressed in sensory neurons, especially ASIC3 [21,22].

Electroacupuncture (EA) belongs to traditional Chinese medicine (TCM). It has often been used to treat dementia induced by stroke [23], polycystic ovary syndrome [24], and pain [25]. Acupuncture analgesia is widely accepted. Recent studies show that the analgesic effect of acupuncture is mediated by the release of endogenous opiates [26], serotonin [27], and adenosine [28]. Low frequency electroacupuncture can induce enkephalin and adenosine release to activate opioid μ receptors and adenosine A1 receptors, respectively. In contrast, high frequency stimulation can release dynorphins to activate κ receptors [26].

Although the analgesic role of acupuncture is well documented, the detailed mechanisms are still unclear, especially its relationship to ASIC3. The purpose of this study was to identify the role of ASIC3 in acupuncture-mediated analgesia in carrageenan- and CFA-induced inflammation pain. Primary hyperalgesia was induced and investigated with an inflammatory inducer delivered through an intraplantar microinjection. Our results showed that ASIC3 was necessary for both carrageenan- and CFA-induced primary mechanical hyperalgesia. We also found that EA at the Zusanli (ST36) with 2 Hz low frequency stimulation can reduce the pain threshold by decreasing the expression of ASIC3 in peripheral DRG neurons.

## 2. Methods

### 2.1 Animals

CD1 mice at 8 to 12 weeks old were used and kept on a 12 h light-dark cycle with sufficient water and food. The animal behavioral tests were conducted blind with experimental groups. The usage of these animals was approved by the Institute of Animal Care and Use Committee of China Medical University, Taiwan, following the Guide for the use of Laboratory Animals (National Academy Press). The number of animals used and their suffering was minimized.

### 2.2 EA treatment

EA treatment was applied using stainless steel needles which were inserted into the muscle layer to a depth of 2-5 mm at ST36. A Trio-300 (Japan) stimulator delivered electrical square pulses for 20 min with a 100 μs duration and a 2 Hz frequency. The stimulation amplitude was 1 mA. The same treatment was given to nonacupoint (the gluteal muscle) to set as the sham control group.

### 2.3 Animal behavior of mechanical hyperalgesia

Mechanical hyperalgesia behavior was tested at 1, 2, 4, and 7 days after intraplantar injections of carrageenan or CFA. All experiments were performed at room temperature (approximately 25°C) and the stimuli were applied only when the animals were calm but not sleeping or grooming. Mechanical scores were measured by testing the number of responses to stimulation. Five applications of von Frey filaments were delivered. Animals were placed on a plexi wire platform in an acrylic chamber and allowed 1 hr for acclimatization. A von Frey filament of 0.02 g bending force was used as the baseline stimulation. A von Frey filament was applied to each hind paw 5 times, with a 30-sec interval between each application.

### 2.4 Immunohistochemistry

Animals were anesthetized with an overdose of chloral hydrate (400 mg/kg, i.p.) and intracardially perfused with saline followed by 4% paraformaldehyde. L3-L5 DRG neurons were immediately dissected and post-fixed with 4% paraformaldehyde. Post-fixed tissues were then placed in 30% sucrose for cryoprotection overnight, embedded in OCT, rapidly frozen using liquid nitrogen, and stored at -80°C. Frozen sections were cut 15-μm thick on a cryostat and mounted on glass slides. Slides were incubated with blocking solution containing 3% BSA, 0.1% Triton X-100, and 0.02% sodium azide in PBS for 2 hours at room temperature. After blocking, slides were incubated overnight with primary antibodies prepared in a blocking solution at 4°C (rabbit anti-ASIC3, 1:1000, Alomone Lab, Jerusalem, Israel). The secondary antibodies were 6 μM Alexa Flour^® ^donkey-anti-rabbit 594 IgG (Molecular Probes, Carlsbad, CA, USA). The stained DRG neurons were then examined using an epi-fluorescent microscope (Olympus, BX-51, Japan).

### 2.5 Western blot analysis

DRG neurons were immediately excised to extract proteins. The total protein was prepared by homogenizing the DRGs in cold radioimmunoprecipitation (RIPA) buffer containing 50 mM Tris-HCl pH 7.4, 250 mM NaCl, 1% NP-40, 5 mM EDTA, 50 mM NaF, 1 mM Na_3_VO_4_, 0.02% NaN_3 _and 1 × protease inhibitor cocktail (AMRESCO). The extracted proteins (30 μg per sample assessed by BCA protein assay) were subjected to 8% SDS-Tris glycine gel electrophoresis and transferred to a PVDF membrane. The membrane was blocked with 5% nonfat milk in TBST buffer (10 mM Tris pH 7.5, 100 mM NaCl, 0.1% Tween 20), and incubated with anti-ASIC 3 (1:1000) subtype antibody (Alomone Labs Ltd) in TBST with 1% bovine serum albumin for 1 hour at room temperature. Peroxidase-conjugated anti-rabbit antibody (1:5000) was used as a secondary antibody. The bands were visualized by an enhanced chemiluminescencent substrate kit (PIERCE) with LAS-3000 Fujifilm (Fuji Photo Film Co. Ltd). Where applicable, the image intensities of specific bands were quantified with NIH ImageJ software (Bethesda, MD, USA).

### 2.6 RNA isolation and Real-time PCR

DRGs were dissected 24 hours after treatment and frozen on dry ice. The tissues were stored at -80°C until RNA extraction. RNA was extracted by Multisource Total RNA Miniprep Kit (Axygen Biosciences, Union City, CA, USA) under standard protocol. 600 μg RNA was subjected to reverse-transcription PCR for converting to cDNA. RNA was first mixed with 1 μl 250 ng Oligo dT18 and 1 μl 10 mM dNTP and heated at 65°C water bath for 5 min and then put on ice for 1 min. 4 μl 5 × First-Stand Buffer, 1 μl 0.1 M DTT, 1 μl RNaseOut and 1 μl SuperScript III Reverse Transcriptase (Invitrogen, Carlsbad, CA, USA) were then added to the mixture. The mixture was incubated at 50°C for 1 hour and inactivated at 70°C for 15 minute to complete the reverse transcription. *Asic3 *gene expression was measured using FastStart Universal SYBG Green Master (Roche, Indianapolis, IN, USA) on ABI 7500 Fast Real-Time PCR system (Applied Biosystem, Carlsbad, CA, USA). Reactions were performed in duplicate. *Asic3 *gene expression was normalized to house keeping gene-glyceraldehyde 3-phosphate dehydrogenase (*Gapdh*) in each sample. For *Asic3*, the primer sequences were 5'-CCCTGTGGACCTGAGAACTT-3' and 5'-CTGCTCACC ACTCCTAAGGG-3'. And for *Gapdh*, the primers sequences were 5'-GGAGCCAA ACGGGTCATCATCTC-3' and 5'-GAGGGGCCATCCACAGTCTTCT-3'. Data are shown as relative expression of control groups.

### 2.7 Statistic analysis

All statistic data are presented as the mean ± standard error. Statistical significance between control, inflammation, EA-sham, and EA group was tested using the ANOVA test, followed by a post hoc Tukey's test (*p *< 0.05 was considered statistically significant).

## 3. Results

### 3.1 Carrageenan- and CFA-induced inflammatory hyperalgesia was attenuated using 2 Hz low frequency EA at the ST36 acupoint

**Figure 1:**
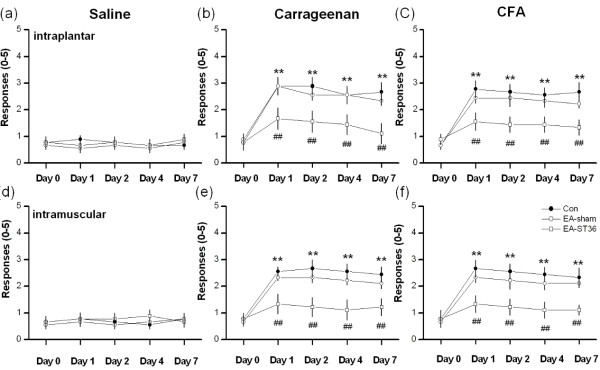
**Primary and secondary inflammation-induced hyperalgesia through carrageenan and CFA injection in paw**. Primary and secondary inflammation-induced hyperalgesia through carrageenan and CFA injection in paw. (a) von Frey filament test in mice from intraplantar injection of saline. (b) von Frey filament test in mice from intraplantar injection of carrageenan. (c) von Frey filament test in mice from intraplantar injection of CFA. (d) von Frey filament test in mice from intramuscular injection of saline. (e) von Frey filament test in mice from intramuscular injection of carrageenan. (f) von Frey filament test in mice from intramuscular injection of CFA. Con: control mice; EA-sham: needles inserted at the ST36 acupoint without electrical stimulation, EA-ST36: needles inserted at the ST36 acupoint with electrical stimulation. *p < 0.05, compared to baseline. ^#^p < 0.05, comparison between Con and EA-ST36 groups. CFA = complete Freund's adjuvant.

To test the mechanical hyperalgesia in inflammation pain, the mechanical stimuli were examined in both ipsilateral and contralateral paws. Figure 1a shows that control, sham, and acupuncture treated mice had a normal response to the von Frey filaments following a saline injection. The saline injection did not induce mechanical hyperalgesia at 7 days after injection in this series experiments (Figure 1a, 0.8 ± 0.2 of control, 0.9 ± 0.2 of EA-sham, 0.8 ± 0.1 of EA, n = 9). After carrageenan injection, mice developed mechanical hyperalgesia with an increase in the number of withdrawals to mechanical stimuli which were applied to the paw ipsilateral to the inflammation side. No hyperalgesia responses were observed on the contralateral sides of the injections (data not shown). These phenomena were also reported in previous studies [7,29]. At 5 applications of the filaments, carrageenan-induced inflammatory paws displayed a hyperactive response on day 1 and maintained it for 7 days. (Figure 1b, solid circle, 2.9 ± 0.3 of day 1 and 2.7 ± 0.4 of day 7, n = 9). To test if EA treatment plays a role in the development of inflammation pain, EA was delivered at the ST36 acupoint at 2 Hz for 20 min. When the mice received the EA treatment, mechanical hyperalgesia attenuated at day 7 after intraplantar carrageenan injection (Figure 1b, 1.1 ± 0.4, open square, n = 8). In the sham group, this did not occur in EA treatment with electrical stimulation at nonacupoint at day 7 (Figure 1b, 2.3 ± 0.2, open circle, n = 9). To make a comparison with carrageenan-induced inflammation pain, EA treatment was investigated to see if it could reverse CFA-induced cell-mediated immune inflammation pain. At 7 days after the intraplantar CFA injection, animals showed an increased withdrawal to mechanical stimulation which indicated mechanical hyperalgesia (Figure 1c, solid circle, 2.7 ± 0.3, n = 9). EA treated mice also displayed a decreased withdrawal to mechanical stimuli, suggesting that it had a curative effect on CFA-induced inflammation pain (Figure 1c, open square, 1.3 ± 0.2, n = 9). Figure 1c shows that these effects were not observed in animals with sham operation (open circle, 2.3 ± 0.3, n = 9). These results significantly reflected the dramatic curative effect of EA treatment in both carrageenan- and CFA-induced primary inflammation pain. Similar results were also observed in secondary pain induced by intramuscular injection. Figure 1d shows that intramuscular injection of saline did not produce hyperalgesia in the control, EA-sham, and ET-ST36 groups. Intramuscular injection of carrageenan induced mechanical hyperalgesia in the control group. This pattern was reversed using EA on the ST36 group (Figure 1e, open square, 1.2 ± 0.2, n = 9) but not on the EA-sham group (Figure 1e, open circle, 2.2 ± 0.2, n = 9). This phenomenon can also been seen in CFA-induced secondary pain. Intramuscular injection of CFA dramatically induced mechanical hyperalgesia which was subsequently abolished with EA at the ST36 acupoint (Figure 1f, open square, 1.2 ± 0.1, n = 9). This did not occur in the sham group (Figure 1f, open circle, 2.2 ± 0.2, n = 9).

### 3.2 The expression of ASIC3 was up-regulated at day 4 after intraplantar carrageenan-mediated inflammation and further down-regulated using 2 Hz EA stimulation using immunohistochemistry staining

**Figure 2:**
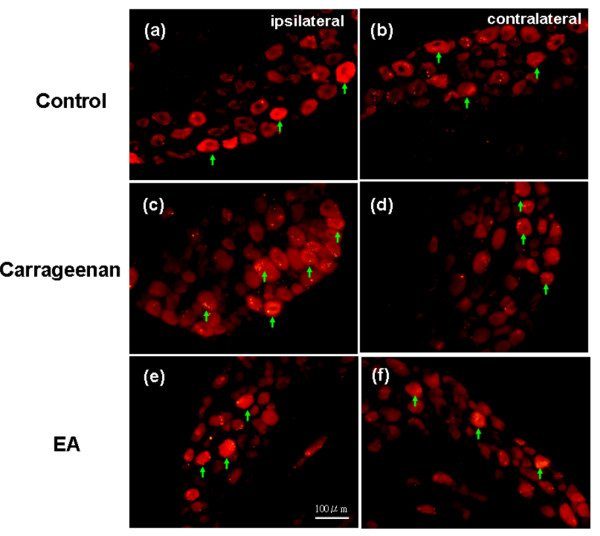
**ASIC3 was increased in lumbar DRGs**. ASIC3 was increased in lumbar DRGs in intraplantar carrageenan-induced hyperalgesia and further attenuated by EA at ST36 acupoint in mice. (a) ASIC3 immunoreactive neurons were found in lumbar DRGs in ipsilateral site of saline-injected group. (b) ASIC3 immunoreactive neurons were found in contralateral site of saline-injected group. (c) ASIC3 immunoreactive neurons were increased in ipsilateral site of carrageenan-induced inflammation. (d) ASIC3 immunoreactive neurons were not increased in contralateral site of carrageenan-induced inflammation. (e) Carrageenan-induced increase of ASIC3 was attenuated by EA in ipsilateral site of inflammation. (f) ASIC3 immunoreactive neurons were not altered by EA in contralateral site of inflammation. Arrows indicated immunoreactive neurons of ASIC3.

The ASIC3 immunoreactivity in the DRG from the control animals was examined to test whether the ASIC3-positive afferents innervating into peripheral tissue sites played a critical role in inflammation. The results showed that ASIC3 was observed in control DRG neurons (Figure 2a). The expression patterns were not significantly different between the inflamed site and the contralateral one (Figure 2b). In the carrageenan-injected mice, the expression of ASIC3 greatly increased, suggesting that ASIC3 participated in carrageenan-induced inflammation (Figure 2c). Otherwise, the carrageenan-induced increase of ASIC3 expression only influenced the ipsilateral site at day 4 after inflammations (Figure 2d). With the use of 2 Hz EA treatment at the ST36 acupoint, the effect of the carrageenan-induced enhancement of ASIC3 expression reduced (Figure 2e). This EA phenomenon was observed in the inflamed site but not the contralateral one (Figure 2f). These results implied that carrageenan-induced inflammation can increase the expression of ASIC3 and that the effect can be further down-regulated by a 2 Hz EA application.

### 3.3 The expression of ASIC3 was up-regulated at day 4 after intraplantar CFA-mediated inflammation and further down-regulated by 2 Hz EA stimulation using immunohistochemistry staining

**Figure 3:**
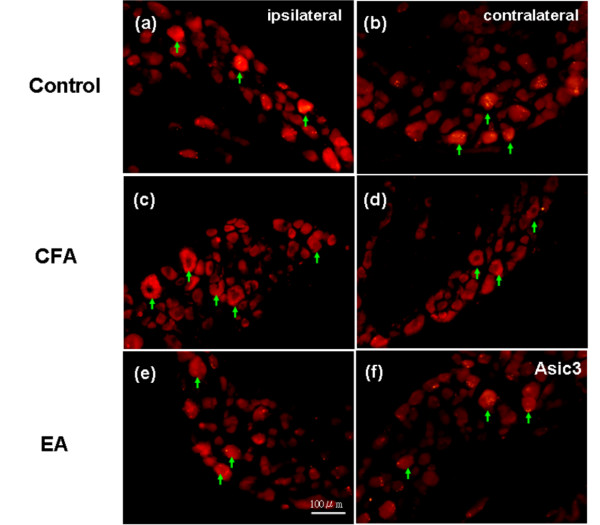
**ASIC3 was increased in lumbar DRGs in intraplantar CFA-induced hyperalgesia**. ASIC3 was increased in lumbar DRGs in intraplantar CFA-induced hyperalgesia and decreased by EA at ST36 acupoint in mice. (a), (b) ASIC3 immunoreactive neurons were found in lumbar DRGs in both ipsilateral and contralateral site of saline-injected group. (c) ASIC3 immunoreactive neurons were increased in ipsilateral site of CFA-induced inflammation. (d) ASIC3 immunoreactive neurons were not increased in contralateral site of CFA-induced inflammation. (e) CFA-induced increase of ASIC3 was attenuated by EA in ipsilateral site of inflammation. (f) ASIC3 immunoreactive neurons were not altered by EA in contralateral site of inflammation. Arrows indicated immunoreactive neurons of ASIC3.

ASIC3 expression during cell immune mediated inflammation was examined. CFA was intraplantarly injected into the mice paws to identify this issue. To serve as a control, ASIC3 immunoreactivity in DRG was confirmed in saline-injected sham control animals. The results showed that ASIC3 was exited in control DRG neurons (Figure 3a). The expression patterns were not significantly different in the injected or contralateral site (Figure 3b). In CFA-inflamed mice, the expression of ASIC3 greatly increased. The result suggested that ASIC3 was involved in CFA-induced inflammation (Figure 3c). In contrast, the CFA-mediated enhancement of ASIC3 expression was only observed in the ipsilateral site but not the contralateral site (Figure 3d). With the use of 2 Hz EA treatment, the effect of the CFA-induced enhancement of ASIC3 expression was reduced (Figure 3e). This EA phenomenon was observed in the injected site but not the contralateral one (Figure 3f). These results suggest that CFA-induced inflammation can increase the expression of ASIC3 and that the phenomenon can be further down-regulated by a 2 Hz EA manipulation.

### 3.4 The quantity of ASIC3 was enhanced by intraplantar carrageenan injection and reversely down-regulated by 2 Hz EA stimulation using western blot

**Figure 4:**
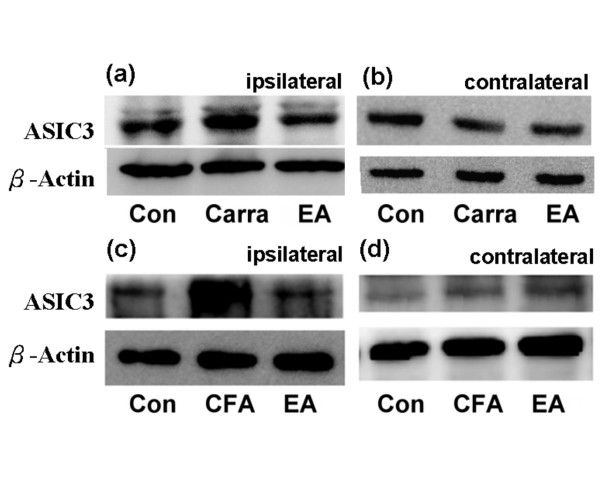
**ASIC3 was increased in lumbar DRGs in both intraplantar carrageenan- and CFA-induced hyperalgesia**. ASIC3 was increased in lumbar DRGs in both intraplantar carrageenan- and CFA-induced hyperalgesia and further attenuated by EA at ST36 acupoint in mice. (a) ASIC3 proteins were increased in ipsilateral site of carrageenan-mediated inflammation and ameliorated by EA at ST36 acupoint (b) ASIC3 proteins were not changed in contralateral site of carrageenan-mediated inflammation and EA stimulation. (c) ASIC3 proteins were increased in ipsilateral site of CFA-induced inflammation and attenuated by EA at ST36 acupoint (d) ASIC3 proteins were not altered in contralateral site of CFA-mediated inflammation and EA stimulation.

To determine if the ASIC3 protein quantity was consistently upregulated with inflammation, western blot was employed. In the control group, ASIC3 proteins were normally expressed in both ipsilateral (Figure 4a, left panel, n = 9) and contralateral DRG neurons (Figure 4b, left panel, n = 9). ASIC3 proteins consistently increased with carrageenen-induced inflammation (Figure 4a, middle panel, 110.8 ± 0.1% compared with control group, n = 9, p < 0.05). These patterns were not observed in the contralateral site (Figure 4b, middle panel, 98.6 ± 0.1% compared with control group, n = 9, p > 0.05). The increased expression of ASIC3 proteins could be down-regulated with a 2 Hz electrical stimulation at the ST36 acupoint at the inflammation site (Figure 4a, right panel, 99.4 ± 3.1% compared with inflamed group, n = 9, p < 0.01) but not at the contralateral site (Figure 4b, right panel, 98.9 ± 2.4% compared with inflamed group, n = 9, p > 0.05). Low frequency electrical stimulation at the ST36 acupoint decreased carrageenan-induced high levels of ASIC3 expression. Similar results were also observed in CFA-induced inflammation. In the controls, ASIC3 existed in both ipsilateral (Figure 4c, left panel, n = 9) and contralateral DRG neurons (Figure 4d, left panel, n = 9). ASIC3 increased in the CFA-injected group (Figure 4c, middle panel 137.1 ± 2.2% compared with control group, n = 9, p < 0.01). These results were not found in the contralateral site (Figure 4d, middle panel, n = 9, p > 0.05). The increased expression of ASIC3 was down-regulated by a 2 Hz EA at the ST36 acupoint in the ipsilateral (Figure 4c, right panel, 97.4 ± 3.7% compared with inflamed group, n = 9, p < 0.01), but not the contralateral, site (Figure 4d, right panel, 98.3 ± 2.4% compared with inflamed group, n = 9, p > 0.05). These results demonstrated that low frequency EA at the ST36 acupoint can successfully reverse inflammation-induced ASIC3 overexpression in both the carrageenen and CFA-induced models.

### 3.5 The expression of ASIC3 was increased by intraplantar carrageenan and CFA injection and reversely down-regulated by EA manipulation using Real-time PCR

**Figure 5:**
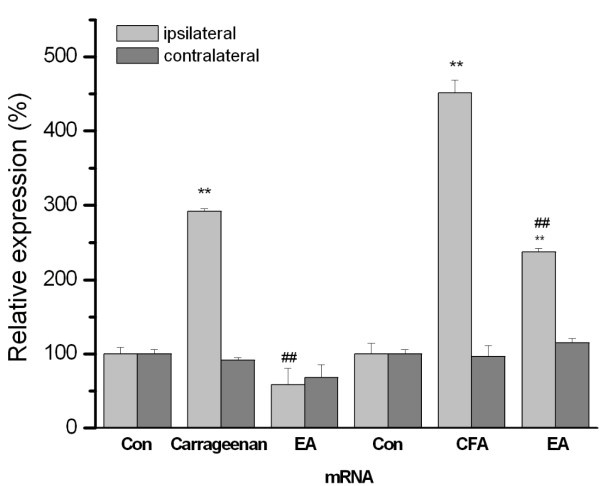
**Comparative quantitative Real-time PCR analysis for ASIC3 mRNAs from control**. Comparative quantitative Real-time PCR analysis for ASIC3 mRNAs from control, inflamed and EA groups. ASIC3 mRNA was reliably increased in lumbar DRGs in both carrageenan- and CFA-induced inflammation pain and further reduced by EA at ST36 acupoint in mice. There is an only unilateral increase in ASIC3 mRNA 24 hours after carrageenan or CFA-induced inflammation. **Significantly greater than control mice (p < 0.01). ## Significant difference compared with inflamed mice (p < 0.01).

We used Real-time PCR to further test if the ASIC3 mRNA quantity was consistently increased with inflammation. In control, ASIC3 mRNA was normally and similarly existed in both ipsilateral (Figure 5, 100 ± 8.8%, n = 3) and contralateral DRG neurons (Figure, 100 ± 5.8%, n = 3). ASIC3 mRNA was increased with carrageenen-induced inflammation (Figure 5, 291.7 ± 3.3% compared with control group, n = 3, p < 0.01). Similar results were not observed in the contralateral site (Figure 5, 91.4 ± 5.3% compared with control group, n = 3, p > 0.05). The enhanced expression of ASIC3 mRNA could be further down-regulated with a 2 Hz EA at the inflammation site (Figure 5, 58.8 ± 22.1% compared with control group, n = 3, p > 0.05) but not at the contralateral site (Figure 5, 68.3 ± 17.3% compared with control group, n = 3, p > 0.05). Low frequency EA ameliorated carrageenan-induced increase of ASIC3 expression. Similar phenomena were also observed in CFA-induced inflammation. In control, ASIC3 was expressed in both ipsilateral (Figure 5, 100 ± 4.4%, n = 3) and contralateral DRG neurons (Figure 5, 100 ± 6.2%, n = 3). The mRNA was increased in the CFA-injected group (Figure 5, 451.5 ± 17.2% compared with control group, n = 3, p < 0.01). These phenomena were not observed in the contralateral site (Figure 5, 96.3 ± 15.1%, n = 3, p > 0.05). The enhanced expression of ASIC3 mRNA was decreased by low frequency EA in the ipsilateral (Figure 5, 237.6 ± 4.5% compared with inflamed group, n = 3, p < 0.01), but not the contralateral site (Figure 5, 115.1 ± 7.9% compared with inflamed group, n = 3, p > 0.05). The above results suggested that 2 Hz EA at the ST36 acupoint can reliably reduced inflammation-induced ASIC3 mRNA overexpression in both the carrageenen and CFA-induced models.

## 4. Discussion

In the current study, we first established an animal model of inflammatory pain with a microinjection of carrageenan and CFA. Animals with inflammatory pain showed mechanical hyperalgesia using a von Frey filament test. Stimulation with a 2 Hz EA at the ST36 acupoint successfully reduced inflammatory hyperalgesia in both carrageenan and CFA groups. ASIC3 expression increased with inflammatory hyperalgesia and was then down-regulated with a 2 Hz EA stimulation, as observed with immunohistochemistry staining and Western blot technique. We further examined the physiological function of ASIC3 using a whole cell recording technique. Our results showed that both the amplitude and percentage of ASIC3-like currents were potentiated in inflammatory hyperalgesia and decreased in the EA group. These phenomena revealed the crucial role of ASIC3 in inflammatory hyperalgesia and the therapeutic role of EA at the ST36 acupoint.

Recent studies have shown that injections of inflammatory inducers result in Nav, TRPV1 and ASIC3 overexpression in DRG neurons [2,30,31]. ASIC3 in primary afferent fibers respond to mechanical hyperalgesia of the paw after inflammatory inducer or repeated acid injection in GM [7,29]. Intramuscular injection of non-selective ASIC blocker amiloride or selective blocker A-317567 prevents mechanical hyperalgesia in mice [2,29]. Interestingly, in the ASIC3 ^-/- ^mice model, ASIC3 is not necessary for thermal pain sensation since deletion of the ASIC protein does not alter its function. The number of ASIC3 channels greatly increased simultaneously in both primary and secondary hyperalgesia. This phenomenon indicated that attenuated ASIC3 overexpression is a potential tool for hyperalgesia formation. Here we reported that a 2 Hz EA at the ST36 acupoint can reduce carrageenan- and CFA-induced inflammatory pain through attenuating ASIC3 overexpression in peripheral DRG neurons.

Previous studies have shown that transcutaneous electrical nerve stimulation (TENS) has the ability to reduce secondary mechanical hyperalgesia of the paw induced by knee joint inflammation [32,33]. Recently, Vance and colleagues have shown that primary mechanical hyperalgesia induced by joint inflammation can be treated by both low- and high-frequency TENS. The hyperalgesia withdrawal threshold was attenuated at 24 hours and 2 weeks after inflammation but not 4 hours. This suggested that the effect of TENS on inflammatory hyperalgesia operates in a time-dependent manner [34]. For TENS, electrodes are often applied to the injury site. TENS is effective but only for a short time. The patient must continue receiving treatment for several days. Recent studies have also shown that giving either low- or high-frequency TENS on the ipsilateral or contralateral sides can reduce hyperalgesia [32,35]. Both low and high frequency TENS-induced analgesia was attenuated in α2A mutant mice. This phenomenon was also observed with the application of α2 AR-selective antagonist at the peripheral level [33]. Peripheral opiate release is also involved in analgesia produced by low frequency TENS. Blockade of μ-opioid receptors prevents the curative effect of low, but not high, frequency TENS [32].

The role of the peripheral and central opioid system in attenuating inflammatory pain has been well-studied [26]. Intraplantar injection of opioid receptor antagonist naloxone can successfully reverse the analgesic effect of EA treatment [36]. Recently, blockage of β-endorphin and corticotropin-releasing factor (CRF) also reduced EA analgesia. A 2 Hz low frequency EA induces the release of enkephalin, while a 100 Hz high frequency EA increases the release of dynorphin in the rat [26]. This result can also be seen in humans [37]. Goldman et al. reported that adenosine, a neuromodulator which serves an analgesic function, can be released through acupuncture to relieve inflammation and neuropathic pain. The curative effective of adenosine requires A1 receptor activation, since this phenomenon cannot be observed in mice lacking A1 receptors [28]. Direct application of an A1 receptor agonist reduced pain sensation. Similar results can be seen by inhibiting enzymes involved in adenosine degradation. The above studies have shown that the opioid and adenosine systems may participate in an analgesic role in both manual and electro-acupuncture. In the current study, we found that ASIC3, one of the most sensitive channels in peripheral inflammatory pain, also modulates the hyperalgesia process and can be regulated by a 2 Hz EA stimulation. ASIC3 mediates the effects of the 2 Hz EA. Interfering with down-regulation of ASIC3 expression may prolong the clinical benefit of EA.

## 5. Conclusions

In summary, we tested the changes in the behavior of ASIC3 protein expression and ASIC3 currents after inducing inflammation with carrageenan and CFA. Mechanical hyperalgesia features were observed in both carrageenan and CFA inflammation models. The mice which received 2 Hz electroacupuncture showed more analgesic reactions to inflammation than those with carrageenan- and CFA-inflammation. We, thus, further examined the ASIC3 protein expression using immunohistochemistry and western blot analysis. Our results showed that ASIC3 greatly increased in both carrageenan- and CFA-induced inflammation. This phenomenon was significantly reversed with a 2 Hz EA at the ST36 acupoint. These findings suggest that ASIC3 is involved in inflammatory hyperalgesia and that the traditional EA acupoint has a curative effect in these inflammation models.

## Competing interests

The authors declare that they have no competing interests.

## Authors' contributions

WHC and CPH performed animal pain behavior WHC and TJL performed immunocytochemistry and western blot. CLH, JTCT and YWL designed the experiment. CLH, TYH and YWL prepared the manuscript and oversaw the research. All authors have read and approved the final manuscript.
